# Fine-Mapping and Genetic Analysis of the Loci Affecting Hepatic Iron Overload in Mice

**DOI:** 10.1371/journal.pone.0063280

**Published:** 2013-05-10

**Authors:** Xin Guo, Zhuzhen Zhang, Fan Zhang, Yunlong Tao, Peng An, Qian Wu, Chia-Yu Wang, Mitchell D. Knutson, Fudi Wang

**Affiliations:** 1 Key Laboratory of Nutrition and Metabolism, Shanghai Key Laboratory of Pediatric Gastroenterology and Nutrition, Institute for Nutritional Sciences, Shanghai Institutes for Biological Sciences, Chinese Academy of Sciences, Graduate School of the Chinese Academy of Sciences, Shanghai, China; 2 Department of Nutrition, Institute of Nutrition and Food Safety, School of Public Health, Collaborative Innovation Center for Diagnosis and Treatment of Infectious Diseases, Zhejiang University, Hangzhou, P.R. China; 3 Department of Food Science and Human Nutrition, University of Florida, Gainesville, Florida, United States of America; University of Turin, Italy

## Abstract

The liver, as the major organ for iron storage and production of hepcidin, plays pivotal roles in maintaining mammalian iron homeostasis. A previous study showed that Quantitative Trait Loci (QTLs) on chromosome 7 (Chr7) and 16 (Chr16) may control hepatic non-heme iron overload in an F2 intercross derived from C57BL/6J (B6) and SWR/J (SWR) mice. In this study, we aimed to validate the existence of these loci and identify the genes responsible for the phenotypic variations by generating congenic mice carrying SWR chromosome segments expanding these QTLs (D7Mit68-D7Mit71 and D16Mit125-D16Mit185, respectively). We excluded involvement of Chr7 based on the lack of iron accumulation in congenic mice. In contrast, liver iron accumulation was observed in Chr16 congenic mice. Through use of a series of subcongenic murine lines the interval on Chr16 was further fine-mapped to a 0.8 Mb segment spanning 11 genes. We found that the mRNA expression pattern in the liver remained unchanged for all 11 genes tested. Most importantly, we detected 4 missense mutations in 3 candidate genes including Sidt1 (P172R), Spice1(R708S), Boc (Q1051R) and Boc (S450-insertion in B6 allele) in the liver of SWR homozygous congenic mice. To further delineate potential modifier gene(s), we reconstituted seven candidate genes, *Sidt1*, *Boc*, *Zdhhc23*, *Gramd1c*, *Atp6v1a*, *Naa50* and *Gtpbp8*, in mouse liver through hydrodynamic transfection. However, we were unable to detect significant changes in liver iron levels upon reconstitution of these candidate genes. Taken together, our work provides strong genetic evidence of the existence of iron modifiers on Chr16. Moreover, we were able to delineate the phenotypically responsible region to a 0.8 Mb region containing 11 coding genes, 3 of which harbor missense mutations, using a series of congenic mice.

## Introduction

Iron has an essential function in mammalian metabolism [1] as it is a functional component of hemoglobin, myoglobin, cytochromes, and other non-heme enzymes involved in oxygen transport, ATP generation, and detoxification. Free iron is also a potent oxidant that promotes the formation of toxic radicals. Consequently, iron metabolism is tightly regulated through complex regulatory mechanisms at both cellular and systemic levels. Systemic iron balance is accomplished through the regulation of iron absorption in the intestine, recycling in the spleen, and storage in the liver [2]. Dysregulation of iron metabolism causes common diseases in mammals that are characterized by iron status; iron-overload is a result of genetic hemochromatosis and iron depletion causes iron-deficiency anemia.

The liver is important for systemic iron homeostasis. It is the major organ for iron storage, and iron within the liver can be mobilized to meet the demands of other organs [3]. Furthermore, hepcidin, the major iron regulator that controls iron absorption in the intestine, is produced mainly in the liver and secreted into circulation [4]. Although many critical discoveries have been made regarding the regulation of iron homeostasis, gaps still exist in our understanding of the details of iron metabolism [5]. The clinical course of hemochromatosis is highly variable, indicating that genetic modifiers may contribute to the complexity of this disease [6], and these variations could make individuals more or less susceptible to the pathological processes of iron metabolism disorders. Identification of such genetic modifiers may help us better understand disease pathogenesis and provide novel insights into the detailed molecular control of iron homeostasis.

Mice are powerful model organisms for investigating iron-related human genetic diseases [7] as it is possible to perform functional studies on candidate genes under controlled environmental factors with defined genetic crosses. Genome-wide quantitative trait loci (QTL) analysis and forward genetic approaches have been successfully used to identify the genetic loci and specific genes that control iron-related quantitative traits such as tissue iron status and red blood cell parameters [8], [9]. Previous studies showed marked differences in the concentrations of liver and spleen non-heme iron content among inbred mouse strains [10]. C57BL mice have low liver and spleen iron content while SWR/J mice have much higher levels. This difference may partially explain the variance in response to infection and susceptibility to uroporphyria between strains [11]. In the past, positional cloning approaches have been successfully used to identify the potent modifiers controlling the variation of iron metabolism between mouse strains [10]. Many putative QTLs affecting iron status have been found, however, few have been validated and genes responsible for these traits remain to be identified [12], [13], [14]. In a recent study, Grant *et al.* undertook QTL analysis to examine the genetic basis of variability in liver and spleen iron content between C57BL/6J and SWR mice. The results showed that QTLs on chromosomes 2, 7, and 16 control liver non-heme iron content in C57BL/6J×SWR F2 male mice [15]. For two QTLs, on Chr16 and Chr7, the SWR alleles were associated with higher iron levels in male mice. In contrast, for the QTL on Chr2 higher iron levels in both liver and spleen were associated with the C57BL/6J allele. However, the modifier genes controlling these QTLs remain to be identified.

In this study, we used forward genetic approaches to identify the gene(s) accountable for the variability of hepatic iron accumulation based on the previous QTL analysis. Generating a series of congenic mouse models, we validated the previously identified QTL on chromosome 16 and refined it to an 830 Kb interval that contains 11 coding genes. Three genes among these have non-synonymous coding sequence variations, which make them strong candidates for being involved in iron metabolism. Although the *in vivo* functional test did not identify specific candidates, our work represents progress in positional cloning of the novel iron metabolism modifier present on Chr16 in mouse liver.

## Materials and Methods

### Mice and diet

Mice were fed a standard rodent laboratory diet (232 mg iron/kg) from SLRC Laboratory Animal Co. Ltd. All experimental protocols were approved by the Institutional Animal Care and Use Committee of the Chinese Academy of Sciences [16]. For iron-rich diet experiments, 8-week-old mice were fed an iron-rich diet (8.3 g of carbonyl iron per kg) of egg-white–based AIN-76A-diets (Research Diets, Inc., New Brunswick, NJ) for 1 week before sacrificing.

### Development and analysis of chromosome 16 and 7 congenic mice

The B6×SWR F1 progeny were backcrossed to B6 to get N2 progeny that harbor different SWR genomic regions. Congenic strains were then developed by continuous backcrossing of N2 progeny to B6 mice with MIT SSLP markers-assisted tracing of the SWR chromosome 16 segment of interest. During backcrossing, we selected mice containing 20 Mb of SWR on chromosome 16 between markers D16Mit125-D16Mit185. After backcrossing with C57BL/6J for five generations, we consider the congenic mice to have a pure C57BL/6J background and backcrossing continued (N≥9) [10]. The heterozygous mice of the initial Line1 were backcrossed to B6 to generate subcongenic lines. The original chromosome 7 congenic line was developed using the same crossing strategy by tracing the SWR chromosome 7 segment between D7Mit68 and D7Mit71. All iron phenotype analyses were performed using 8-week-old males unless otherwise indicated. Genotyping primers are listed in **Table S1.**

### Genomic DNA sequence analysis

DNA segments of every exon and intron-exon boundary of the candidate genes were amplified using PCR and the sequences were aligned in comparison with B6 using Lasergene software (DNASTAR). All sequencing data has been deposited in GenBank and the Accession numbers are listed in **Table 1**.

**Table 1:** Summary of genomic DNA sequencing results in the candidate interval.

Gene	Accession number	Polymorphism	Position in gene	Protein Variation
*Sidt1*	rs4179590	G/A	Exon3	
	rs4179589	G/C	Exon3	P172R
	rs51960690	G/A	Exon10	
	rs50972129	A/G	Exon10	
	rs4179517	A/G	Intron11–12	
	rs4179515	G/A	Exon12	
	rs48139623	A/G	Intron14–15	
	rs32704753	G/A	Exon16	
	rs4179510	T/C	Exon16	
	rs4179504	A/G	Exon18	
*Spice1*	rs4179702	C/A	Exon14	R708S
	rs31418354	T/C	Exon14	
*Boc*	NW_001030584.1	CGT insertion	Exon8	S450-insertion
	rs4179917	C/A	Intron11–12	
	rs4179913	C/T	Exon16	
	rs45694900	A/G	Exon16	
	rs4179908	T/C	Exon19	Q1051R
	rs4179907	G/A	3′UTR	
*Wdr52*	rs4179728	A/G	Exon3	
	rs4179729	T/C	Exon3	
	rs4179745	T/C	Intron10–11	
	ENSMUSSNP328538	C/A	Intron14–15	
	rs32712110	T/C	Exon17	
	rs4179843	C/T	Intron26–27	
	rs4179872	T/C	Exon28	
	rs31418356	C/T	Intron28–29	
	rs32712642	C/T	Exon31	

### Measurements of tissue non-heme iron, heme, and serum iron

Quantitative measurement of tissue non-heme iron and serum iron parameters were performed as previously described [17]. Heme content was measured using QuantiChrom™ Heme Assay Kit (BioAssay Systems, DIHM-250) according to the manual and was normalized using protein concentrations.

### Quantitative RT-PCR

Quantitative RT-PCR (qPCR) was performed as described previously [16]. Raw data was normalized to internal β-actin and presented as relative expression levels. All primers for qPCR are described in **Table S2**.

### Western blots

Western blots were performed as previously described [16]. Primary antibodies used are as follows: rabbit anti-H-ferritin (1∶1000 dilution, Alpha Diagnostics International), rabbit anti-L-ferritin (1∶1000 dilution, ABCAM), rabbit anti-flag(1∶1000 dilution, cell signaling), rabbit anti-Sidt1(1∶1000 dilution, Santa Cruz), rabbit anti-β-actin (1∶2000 dilution, Sigma), rabbit anti-mouse ferroportin(1∶1000).Quantification of western blots was performed using Quantity One (Bio-Rad) according to the manual.

### Constructs and plasmids

Full-length mouse cDNA of candidates including *Zdhhc23*, *Gramd1c*, *Atp6v1a*, *Naa50*, *Sidt1*, *Boc* and *Gtpbp8* were obtained from Open Biosystems. We first cloned each gene's CDS into pCMV-3Tag-3A (Agilent Technologies) with a C-terminally expressed 3×Flag tag. The 3×flag fusion proteins were then subcloned into the final pLive Vector (Mirus, Madison, WI) for Hydrodynamic-based transfection. For cell-based iron uptake assays, CDS of *mSidt1* (B6 and SWR allele) was cloned into pCMV-Sport6 (Open Biosystems). Mutagenesis: The pCMV-Sidt1-SWR clone containing the P172R mutation was constructed using the QuikChange® II Site-Directed Mutagenesis Kit(Stratagene) following the manufacturer's instructions.

### Hydrodynamic-based transfection *in vivo*

The pLive Vector designed for prolonged expression of transgenes in mouse liver was purchased from Mirus (Madison, WI). All plasmid DNAs were purified with the Endofree Plasmid Maxi Kit (Qiagen). A total of 30 ug purified plasmid DNA was delivered into the livers of 6-week-old C57BL/6J male mice by hydrodynamic tail vein injection of the plasmid in a 10% body volume of saline.

### Measurement of iron uptake

HEK 293T cells were transiently transfected with mSidt1 (B6 and SWR allele), mZip14 [18], or an empty vector for 48 h (Fugene HD, Roche). Prior to uptake, cells were washed twice with serum-free medium (SFM) and incubated at 37°C for 1 h in SFM containing 2% BSA to deplete cells of transferrin and to block nonspecific binding. The uptake buffer (130 mM NaCl, 10 mM KCl, 1 mM CaCl_2_ and 1 mM MgSO_4_) was adjusted to the indicated pH value (7.5, 6.5, and 5.5) using HEPES buffers [19], [20]. For uptake, cells were incubated with 2 µM ^59^Fe-ferric citrate in SFM in the presence of 1 mM L-ascorbic acid for 2 h at 37°C, followed by washing three times with iron chelator solution (1 mM bathophenanthroline sulfonate and 1 m M diethylenetriaminepenta acetic acid) to remove any surface-bound iron. Cells were lysed in buffer containing 0.2 N NaOH and 0.2% SDS. Radioactivity was determined by gamma counting, and protein concentration was determined colorimetrically by using the *RC DC* protein assay (Bio-Rad). Three independent assays were performed in triplicate.

### Statistical analysis

Data are presented as mean ± SEM. All experiments were performed in triplicate. Student's t-test was used for comparison between two groups (unpaired, 2-tailed). *P*<0.05 was considered significant.

## Results

### Validation that the QTL on Chromosome 16 affects basal liver non-heme iron content

**Figure 1:**
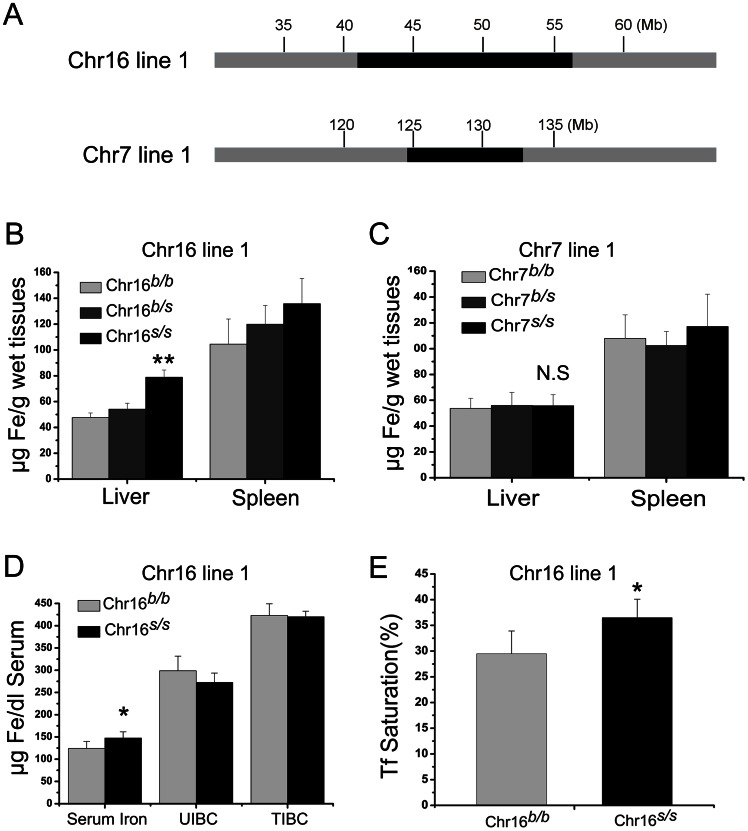
Haplotypes and quantitative measurements of tissue iron in original congenic strains of chromosome 16 and 7. (A) Schematic of original congenic lines carrying portions of SWR chromosomes 7 and 16 (black) on a B6 (grey) background. (B) Liver and spleen non-heme iron concentrations of Chr16 Line 1. (C) Liver and spleen non-heme iron concentrations of congenic lines of Chr7 Line 1. (D) Serum iron, unsaturated iron binding capacity (UIBC) and total iron binding capacity (TIBC), and (E) Serum transferrin saturations (Tf%) measurement of Chr16 Line 1. Data represent mean ± SEM. N.S: no significance, * P<0.05, ** P<0.01. n>5 for each group.

A previous linkage analysis by Grant *et al.* identified two QTLs, one on Chr7 and one on Chr16, affecting basal non-heme iron content in murine liver [21]. The microsatellite markers nearest the calculated peak of the QTLs were identified as D7Mit71 and D16Mit30. To extend on this study, congenic mice were created for Chr7 and Chr16 by producing F1 hybrids between C57BL/6J and SWR, followed by at least nine successive backcrosses to the C57BL/6J parental strain. The genome interval transferred in the original congenic lines spanned from D7Mit68 to D7Mit71 and from D16Mit125 to D16Mit185 (**Figure 1A**). The regions extended beyond the confidence interval to ensure that all the genetic factors that could contribute to the QTL-associated phenotype would be transferred to the resulting congenics.

We used an intercross strategy to determine the iron phenotype of the SWR homozygous (*s/s*) progeny of each congenic mouse at the age of 8 weeks. The Chr16*^s/s^* congenics containing the SWR haplotype between D16Mit125 to D16Mit30 showed significantly higher liver non-heme iron content compared with Chr16*^b/b^* controls, indicating that the previously detected QTL fell in this region (**Figure 1B**). In contrast, the Chr7*^s/s^* congenics containing the chromosome 7 segments did not show significant differences in liver non-heme iron content compared with the Chr7*^b/b^* controls (**Figure 1C**). Moreover, Chr16*^s/s^* congenics also showed significantly higher serum iron levels and transferrin (Tf)-saturation levels compared with Chr16*^b/b^* controls (**Figure 1D, 1E**). No significant changes in iron non-heme content of spleen or other major organs (**Figure S1**) were found in Chr16 congenics. Thus, we chose the Chr16 congenic line for further study.

### Fine mapping of the QTL on Chromosome 16 with congenic mice

**Figure 2:**
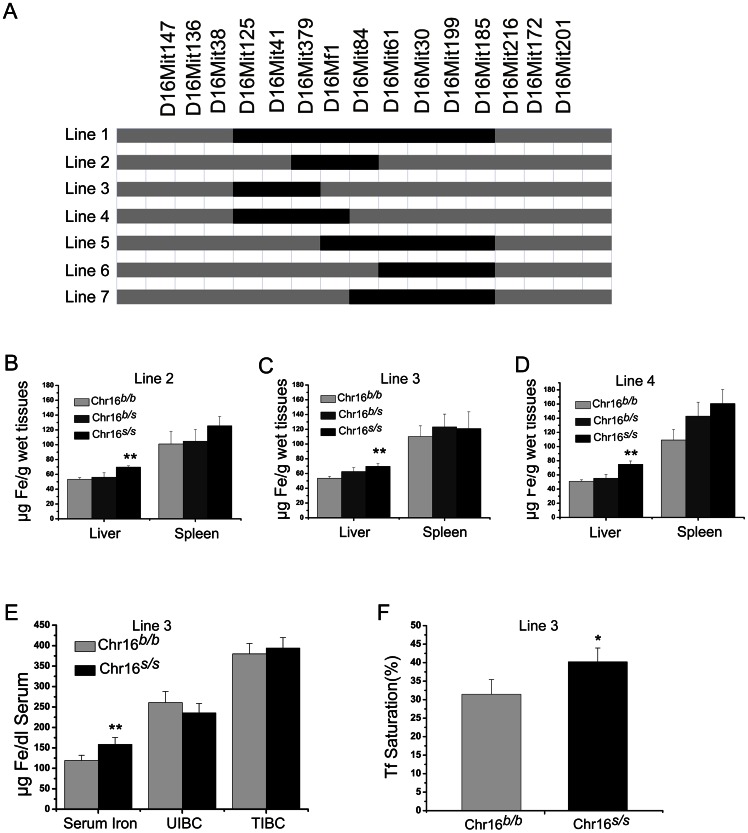
Haplotypes and iron phenotype analysis of subcongenic lines of Chr16. (A) Map of subcongenic lines derived from the original line. (B–D) Measurement of liver and spleen non-heme iron of congenic Line 2, Line 3, and Line 4 (n>5 per group). (E) Serum iron, unsaturated iron binding capacity (UIBC), and total iron binding capacity (TIBC), and (F) Serum transferrin saturation (Tf %) measurement of congenic Line 3. Data represent mean ± SEM. * P<0.05, ** P<0.01.

To fine-map the QTL on Chr16, we continuously backcrossed the initial Chr16 congenic line 1 with B6 mice to generate subcongenic lines carrying various SWR chromosome segments between D16Mit125 and D16Mit185 (**Figure 2A**). Six subcongenic lines were developed successfully. We then examined the iron phenotype of these lines. Out of the six congenic lines, three lines of *s/s* homozygous (Chr16 line 2, line 3, and line 4) showed significant increases in liver non-heme iron content, which were consistent with the original line 1 (**Figure 2B, 2C, 2D**). In contrast, the other three lines of *s/s* homozygous mice (Chr16 line 5, line 6, and line 7) did not show such differences (**Figure S2A, S2B, S2C**).

Congenic line 3 contained the smallest critical region spanning from D16Mit379 to D16Mf1, and thus, was used for further study. The *s/s* homozygous of line 3 also showed significantly higher serum iron and transferrin saturation levels compared with *b/b* homozygous controls (**Figure 2E, 2F**).

### Expression profile of iron-related genes in congenic mice

**Figure 3:**
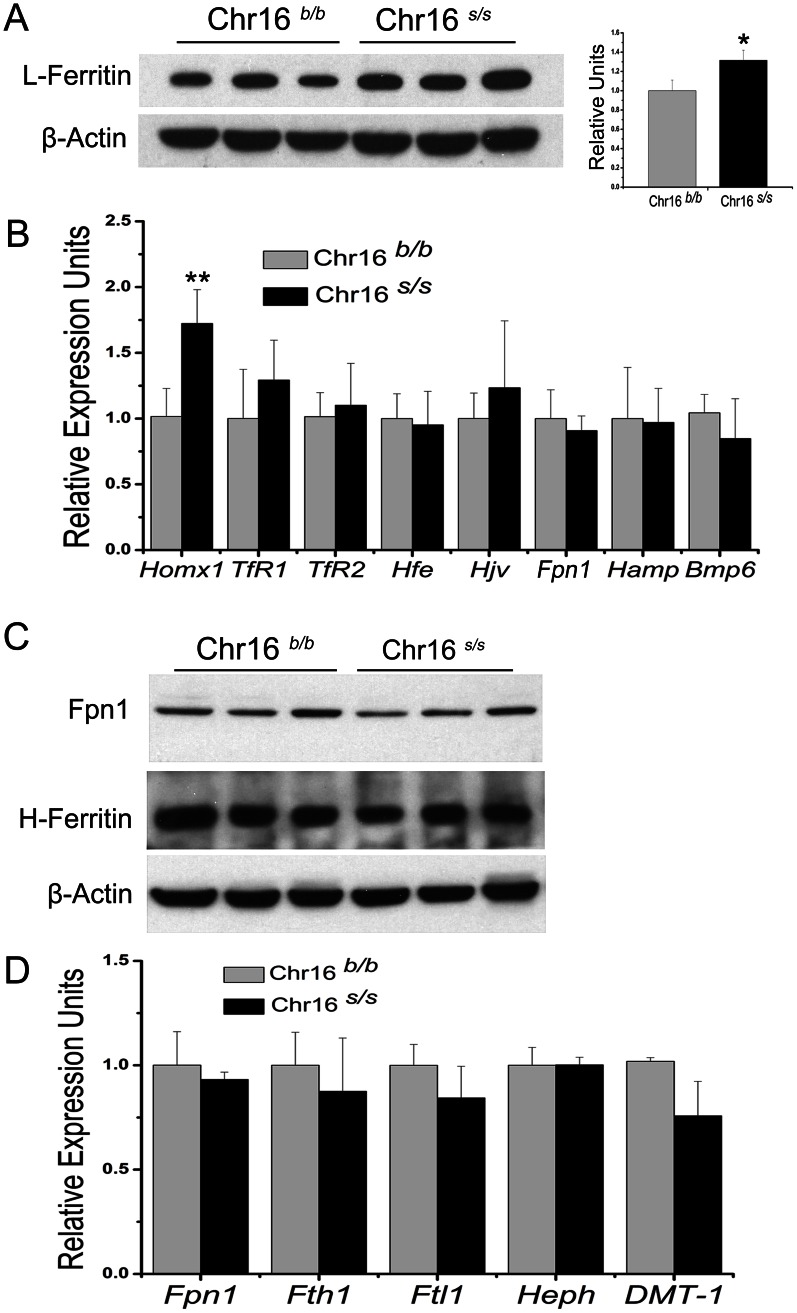
Expression profile of selected iron-related genes in congenic liver and duodenum. Ferritin-L protein expression in liver (A) and ferroportin1 and ferritin-H expression in duodenum (C) of Chr16*^b/b^* and Chr16*^s/s^* mice were assessed by western blot. Quantification of protein levels normalized to actin was performed using densitometry in three independent experiments. Relative mRNA levels of selected iron-related genes were measured by qRT-PCR in liver (B) and duodenum (D) of Chr16 congenics and are represented as ratios normalized to Chr16*^b/b^*, which was defined as 1.0. n> = 5 for each group. Data represent mean ± SEM. * P<0.05, ** P<0.01.

The candidate gene(s) underlying this QTL may affect iron homeostasis through modification of known iron-regulatory pathway(s) and iron-related genes. Thus, we examined the expression of some iron-related genes in the liver and intestine of congenic mice. Consistent with higher hepatic non-heme iron content, the ferritin protein levels, an indicator of intracellular iron levels, were significantly higher in the *s/s* homozygous mice compared with the *b/b* homozygous controls (**Figure 3A**).

The qPCR results showed a 1.7-fold upregulation in the expression of the iron-related gene, *Hmox1*, in Chr16*^s/s^* congenic livers compared with Chr16*^b/b^* control livers, which was consistent with the hepatic iron accumulation (**Figure 3B**). Complex regulatory mechanisms control the expression of *Hmox1*; this protein can be induced by iron, oxidative stress and inflammatory stimuli. In addition, *Hmox1* can be regulated by cellular heme level through the transcription factor Bach1 [22], [23]. We found significant increases in heme content and the expression of *Fth1*, another downstream target of Bach1, in Chr16*^s/s^* congenic livers compared with Chr16*^b/b^* controls. We also detected a significant increase in expression of *Sod1* and *Gpx4*, which are involved in the anti-oxidant response (**Figure S3A, S3B**). In contrast, we found no significant changes in mRNA levels of pro-inflammatory cytokines including *IL-6*, *TNF-α, or IL-1β* in Chr16*^s/s^* congenic liver or spleen (**Figure S3C**) indicating that the inflammatory status in our models is normal. Taken together, we found a significant increase in heme content and oxidative stress level as indicated by the increase in expression of *Sod1* and *Gpx4* in Chr16*^s/s^* liver. It is likely that the increase in iron and oxidative levels contributed to the upregulation in expression of *Hmox1* in Chr16*^s/s^* liver. Furthermore, there were no differences in gene expression of other iron-related genes in livers and spleen from *b/b* and *s/s* mice (**Figure 3B** **and Figure S3D**).

In systemic iron metabolism, intestinal iron absorption can influence hepatic iron content, which reflects whole body iron stores. Thus, we examined the expression of some genes controlling intestinal iron absorption. Western blots showed comparable protein levels of Ferroportin1 (**Figure 3C**), the major iron-exporter, in duodenal enterocytes. H-ferritin is required to maintain intestinal iron efflux and H-ferritin deletion in the intestine increases body iron stores and transferrin saturation [24]. As our congenic models displayed similar iron-related phenotypes, we tested whether there was an H-ferritin expression difference in the duodenum in Chr16*^s/s^* congenics. However, both qPCR and western blot results indicated that there were no differences in H-ferritin expression at the mRNA or protein levels (**Figure 3C**). Additionally, qPCR results showed no significant differences in the expression of selected genes involved in intestinal iron absorption (**Figure 3D**) between Chr16*^s/s^* and Chr16*^b/b^* groups.

### Analysis of the candidate genes through genomic DNA sequencing and mRNA expression profiling

**Figure 4:**
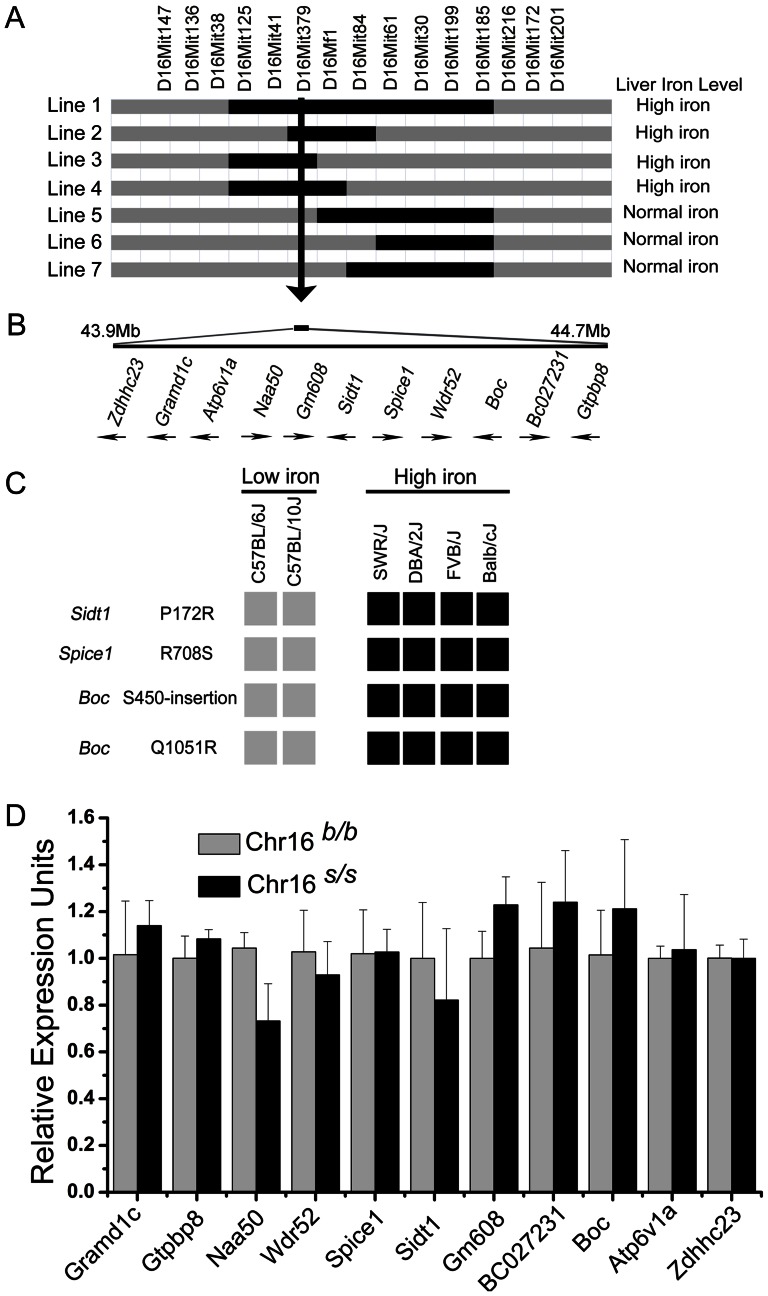
Fine mapping of the QTL on Chr16 and candidate genes in the critical interval. (A) Refinement of the Chr16 QTL location using sublines. Genotyping markers defining the boundaries are indicated across the top. The black portion of the lines indicates homozygosity for SWR alleles while grey indicates the B6 background. Liver iron status was grouped as normal (less than 55 mg/g) or high (higher than 70 mg/g) by an arbitrary cut-off and is shown on the right of each panel. (B) The critical candidate region was localized to an 840 kb region as indicated by the vertical arrow. Arrows indicate the direction of transcription. (C) Haplotype panel of inbred mouse strains for the four non-synonymous SNPs. Genotypes of SNPs were ascertained by direct DNA sequence analysis. Alleles identical to B6 alleles are shown in grey and alleles identical to SWR alleles are shown in black. (D) Relative mRNA expression of candidate genes in Chr16 congenic mouse liver. Expression of candidate genes is reported as relative expression levels using *β-actin* as an internal control in each group. The Chr16^s/s^: Chr16*^b/b^* ratios indicate relative expression levels of the Chr16*^s/s^* group normalized to the Chr16*^s/s^* group, which was defined as 1.0.

Iron phenotype analysis of the congenic lines refined the candidate interval of chromosome 16 to an 830 kb region (**Figure 4A**). The Ensembl database (Ensembl Genes 67) indicated that 11 candidate genes are located within this critical region (**Table S3**). None of these candidate genes have previously been reported to have a direct role in iron metabolism. We speculate that the gene(s) underlying this QTL may either have a coding sequence variation that changes the function of the protein or a regulatory sequence variation that could cause differences in gene expression levels between the two parental strains. Exon sequencing all of 11 genes in the critical interval (**Figure 4B**) identified 27 SNPs across four genes (**Table 1**). Among these, three genes have coding sequence changes: Sidt1 (P172R), Spice1 (R708S), Boc (Q1051R and S450-insertion in B6 allele).

We evaluated the conservation of these four non-synonymous variations by exon sequencing between mouse strains that exhibited differing liver iron statuses. All of the four variations showed consistently different haplotypes between low liver iron strains (C57BL/6J and C57BL/10J [10]) and high liver iron strains (FVB/J, DBA/2J and Blab/cJ) (**Figure 4C**).

*Boc* has an insertion of a Ser residue in C57BL strains that makes it different from all other mouse strains and species tested. Moreover, *Boc* also contained a Q1051R variation, which is conserved in some other mammalian species (**Figure S4A, S4B**).

The P172R amino acid substitution in Sidt1 is unique to C57BL strains, altering a residue that is conserved between mouse strains and primates (**Figure S4C**). While Spice1 has an R708S variation at a poorly conserved residue (**Figure S4D**).

To test whether the expression variations of these candidates contributes to the QTL, we examined the expression profile of all 11 candidates in congenic mouse livers using qPCR. None of the investigated genes exhibited significant differences in their mRNA expression levels in liver, intestine, or spleen in Chr16*^s/s^* mice compared with Chr16*^b/b^* controls (**Figure 4D** **, Figure S5A, S5B**). To Further investigate the possible involvement of these candidates in iron metabolism, we performed expression profiling of candidates in livers of mice treated with an iron-rich diet. After mice were fed a high iron diet for 1 week, we detected a 10-fold upregulation of hepatic non-heme iron content (data not shown) and significant decrease in *TfR1* expression. We found a significant increase in expression of *Naa50* and *Gtpbp8* and significant decrease in expression of *Zdhhc23* in livers of mice with iron overload (**Figure S5C**). The expression of other candidates was not affected.

### Overexpression and functional testing of candidates *in vivo*

**Figure 5:**
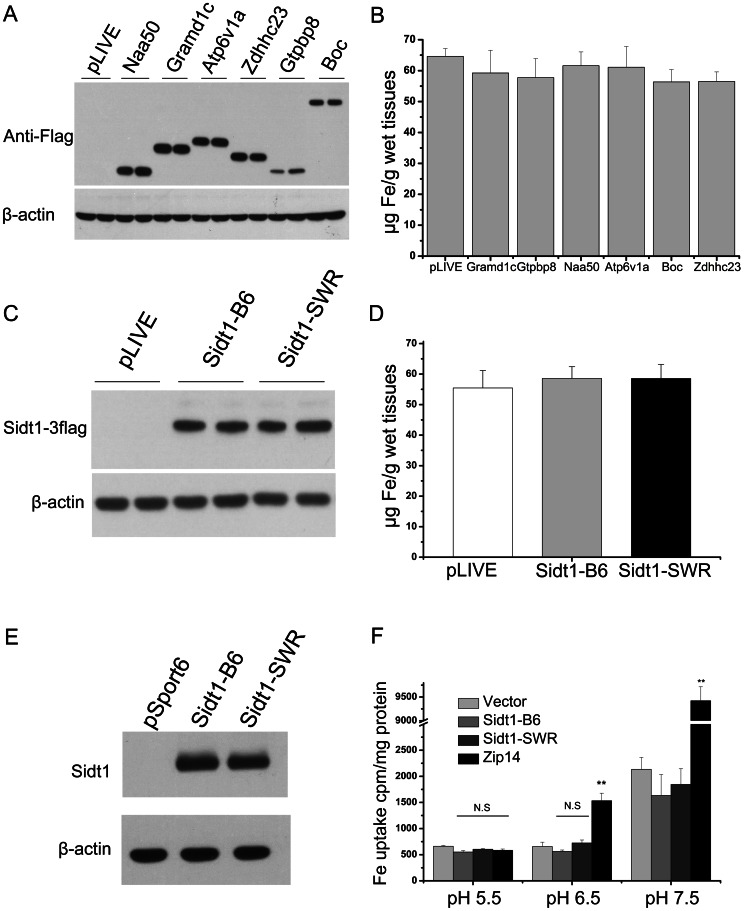
Overexpression of candidate genes in mouse liver and iron phenotype analysis. Candidate genes were delivered to the mice through hydrodynamic transfection. Four weeks after injection, livers were subjected to analysis (n = 5 male mice for each group). (A) Overexpression of candidate genes in mouse liver was examined by western blot. Each lane represents an individual mouse in each group. (B) Liver non-heme iron content of each group was examined (n = 5 male mice for each group). (C) Overexpression of mSidt1-B6 and mSidt1-SWR in mouse liver was examined by western blot. (D) Liver non-heme iron content of each group was measured (n = 5 male mice for each group). (E,F) Analysis of mSidt1(B6 and SWR alleles) in an iron uptake assay. HEK 293T cells were transiently transfected with empty vector, mSidt1-B6, mSidt1-SWR, and mZip14. 48 h after transfection, cells were harvested and 20 ug of protein was used for western blot analysis to verify expression of mSidt1 (B6 and SWR). Radioactivity was detected by gamma counter and normalized using protein concentrations. Similar results were obtained in three independent experiments with triplicates. Data represent mean ± SEM. N.S: no significance, * P<0.05, ** P<0.01.

As expression profiling analysis did not identify a candidate(s) that could be used for a functional study, we aimed to overexpress each candidate in the liver to test their possible function in iron metabolism. Hydrodynamics-based transfection of the liver is a rapid delivery approach with which to study novel gene function *in vivo*. In fact, this well-established technique has been previously shown not only to successfully deliver expression constructs to the liver with high efficiency, but also to maintain long-term expression of target genes [25]. For this purpose, we generated expression constructs of six candidates with previously identified specific biological functions and successfully expressed them in C57BL/6J wild-type mouse liver [26], [27], [28], [29], [30], [31]. Six-week-old C57BL/6J mice were injected with 30 µg of construct DNA via hydrodynamic tail vein injection. Four weeks after injection, the expression of candidates in the liver was confirmed by western blot (**Figure 5A**). We then analyzed the iron phenotype of these mice. None of the six candidates showed the ability to alter hepatic non-heme iron content after successful overexpression in liver (**Figure 5B**). Further, we did not detect a significant change in expression of iron-related genes (**Figure S6**) or in serum iron or transferrin saturation (data not shown).

Among the candidates, *Sidt1* encodes a mouse gene homologous with Systemic *RNAi* Deficient-1 in *C. elegans*, a multi-span transmembrane protein necessary for systemic *RNAi* activity [32]. A recent study identified *Sidt1* as an active dsRNA-gated dsRNA channel [33]. In mice, Sidt1 protein is located on hepatocyte plasma membranes [34]. Furthermore, sequence analysis and biochemical experiments suggest that *Sidt1* proteins are composed of an N-terminal extracellular domain followed by 9–12 predicted transmembrane regions, and that the extracellular domain controls its intrinsic ability to transport dsRNA [35]. The P172R variation detected between B6 and SWR mice is located in the extracellular N-terminus of mSidt1. We speculated that this variation may affect protein function and thus selected Sidt1 as a strong candidate for this study. Hydrodynamics-based transfection was used to test the function of mSidt1 (B6 and SWR alleles) in the mouse liver. However, after successful overexpression of the two proteins in mouse liver (**Figure 5C**), no significant changes in liver iron were found compared with empty vector controls (**Figure 5D**). We next attempted a cell-based *in vitro* functional assay to look for evidence of the possible involvement of this molecule in iron metabolism. Bioinformatics predicts that Sidt1 may function as a transmembrane cation metal ion transporter [36]. We performed a non-transferrin-bound iron (NTBI) uptake assay to test whether mouse Sidt1 harbors the ability to function as an iron transporter. HEK 293T cells were transiently transfected with mSidt1-B6, mSidt1-SWR, mZip14, or empty vector. Western blot analysis revealed successful expression of Sidt1 protein (**Figure 5E**). However, overexpression of *mSidt1* (both B6 allele and SWR allele) did not increase the uptake of NTBI (**Figure 5F**) at different pH values. In all assays, mZip14 was included as a positive control [18]. Moreover, a transferrin-bound iron (TBI) uptake assay showed that mSidt1 did not mediate TBI uptake either (data not shown). Thus, we conclude that mSidt1 does not directly mediate iron uptake into cells.

## Discussion

For studies of susceptibility and phenotypic variations in many diseases, characterization of the genetic architecture and identification of the genes that act as modifiers of complex traits are important, but difficult, studies. Systemic iron homeostasis is a complex trait influenced by multiple genetic factors. QTL mapping accompanied by positional cloning has been used previously to identify novel genes involved in iron homeostasis. In fact, many putative QTLs have been identified by classic intercrosses of different mouse strains with varied iron statuses [12], [13], [14]. However, it is believed that the true number of QTLs is smaller than reported [37] as few have been validated or fine-mapped through use of congenic mouse models.

In our study, we provided evidence to verify the QTL previously identified on Chr16, which is responsible for the difference in basal hepatic iron levels between B6 and SWR mice. The QTL was then refined from a 20 Mb interval down to a 0.8 Mb interval containing only 11 genes. Although we failed to verify any candidate genes specifically involved in systemic iron homeostasis, our study still represents a step forward in positional cloning of this QTL and provides a foundation for further identification of the novel functions of these genes in iron metabolism.

We first investigated these candidates using genomic DNA sequencing and mRNA expression profiles. We then performed an unbiased functional screen on several candidates using hydrodynamic-based overexpression in the liver. While the overexpression of candidate genes was successful, none of the seven candidates tested altered liver iron status. This method is both rapid and relatively high-throughput making it an ideal assay for our study; however, its range is somewhat limited. It is possible that candidate genes may function in organs other than liver to influence liver iron status, considering that both the serum iron and transferrin saturation are high in Chr16*^s/s^* congenics. Thus, liver-specific overexpression of the candidates may not reveal function. The knockout mouse approach may prove to be a better strategy by which to study these candidate genes, and we plan to employ this tactic in future studies.

In some cases, QTLs may result from several polymorphisms residing in one gene or closely linked genes rather than a single genetic variation [38], [39], [40]. In our study, we have identified a total of 27 polymorphisms in four candidates (**Table 1**). However, it would be prohibitively laborious to perform functional tests on each candidate variation as well as their different combinations.

Among the candidate genes, three have non-synonymous coding sequence variations. *Sidt1* has a conserved P172R variation represented in C57BL strains (Pro). Previous studies indicated that Sidt1 functions as an active dsRNA channel [34] and bioinformatics analysis suggested that Sidt1 may possess iron transporter activity [41]. Thus, we selected Sidt1 for our first functional study. Two forms of Sidt1 (B6 and SWR alleles) were overexpressed in liver but failed to change hepatic iron content. A cell transfection assay also ruled out the possibility that mouse Sidt1 acts as a direct NTBI transporter. However, it is possible that Sidt1 could uptake some other solutes and in turn, affect iron homeostasis. Future studies will be required to assess this possibility.

*Boc* has two non-synonymous variations, with the Ser450 insertion variation being highly conserved between mouse strains and other species making it a relatively strong candidate. However, *Boc* gene expression in the liver is low and its only reported function is during neural development [26], [42]. Considering this, overexpression of *Bo*c in the liver may not fully reflect its role *in vivo*. The available *Boc* knockout mouse model should provide a superior method with which to assess *Boc*'s possible role in iron metabolism [26]. *Spice1* also has a non-synonymous variation. However, as a novel gene without validated *in vivo* functions, it is hard to predict its possible role or mechanism of action in iron metabolism.

Among the candidates genes, *Atp6v1a* is reported to be involved in iron metabolism. *Atp6v1a*, ATPase H^+^ transporting lysosomal V1 subunit A, belongs to the V-type ATPase protein family. This protein complex is responsible for pumping protons from the cytoplasm to the lumen of organelles and plays important roles in endosomal acidification, which is important for the endocytic pathway [28]. V-type ATPase inhibitors have been shown to affect transferrin recycling and iron uptake [43], [44]. Additionally, Nieland, *et al.* found two sulfonamide compounds that could reversibly inhibit transferrin-mediated iron uptake through alteration of endosomal and lysosomal pH by inhibiting V-ATPase's ATP hydrolysis activity [45]. The zebrafish (*Danio rerio*) orthologue of *Atp6v1a* has a critical role in embryonic acid secretion and ion balance [46]. Together these findings strongly implicate *Atp6v1a* in iron metabolism; however, we did not find any coding sequence variations or expression differences in this gene in our congenic mouse models. Nonetheless, the possibility remains that there are variations in Atp6v1a protein levels, including protein content and subcellular localization, in our models. Further studies are necessary for detailed investigation of Atp6v1a's role in iron metabolism.

Using the iron rich diet-treated mouse model, we detected 3 genes (*Zdhhc23, Naa50 and Gtpbp8*) with changes in expression induced by severe hepatic iron overload. Among these genes, *Zdhhc23* (zinc finger, DHHC domain containing 23) is a palmitoyl-transferase involved in protein palmitoylation. *Zdhhc23* is reported to control the palmitoylation of calcium-activated potassium channels, which is important for the surface expression of these ion channels [27]. Also, palmitoylation is important for cell-surface expression, spatial organization, and activity of many other ion channels [47]. The decreased expression of *Zdhhc23* in iron overload liver may represent a response to iron accumulation by regulation of the activities of ion channels that could potentially be responsible for iron metabolism. Whether these genes are involved in iron metabolism will require further study.

Hemochromatosis is a typical iron-related disorder characterized by excessive iron-overload in the liver and other parenchymal organs. Genetic mutations affecting *hamp1* expression or the hepcidin-Ferroportin1 axis can cause this syndrome. In our congenic models, we found that the expression of *hamp1* is normal and the signaling pathways controlling hepcidin expression examined (BMP-HJV, TfR2-HFE, and inflammation pathways) are intact. We also did not find gene expression changes in *Fpn1*. Thus, we inferred that the candidate modifier(s) may affect liver iron homeostasis through pathways other than hepcidin regulation.

We found a significant increase in both liver and serum iron levels in Chr16*^s/s^* congenics, which implied co-upregulation of these traits by the candidate genes [37]. Moreover, this indicated the causal link between upregulation of intestinal iron absorption and elevated hepatic iron content. Although we did not find significant changes in the expression levels of genes involved in iron absorption (DMT-1, Dcytb, Hephestin, Ferroportin1 and H-Ferritin) between Chr16*^s/s^* congenics and Chr16*^b/b^* controls, we still could not rule out the possibility that changes in intestinal iron absorption may partially contribute to the elevated hepatic iron accumulation. More comprehensive approaches including intestinal radioactive iron absorption assays are needed to fully investigate this possibility.

Mouse heme oxygenase 1 (*Hmox1*) catabolizes cellular heme to biliverdin, carbon monoxide, and free iron [48]. This process is crucial for the expulsion of iron from tissue stores. *Hmox1* KO mice showed low serum iron and anemia while they had mild iron loading in liver and kidney at 50 weeks of age [49]. *Hmox1* expression is regulated by heme, iron, oxidative stress and inflammatory stimuli [22]. In our model, we concluded that the upregulation of *Hmox1* expression in congenic liver is induced by elevated iron and heme content and oxidative stress level.

The differences in hepatic iron status in these mice could contribute to predisposition to iron-related disorders. SWR mouse strains are more susceptible to uroporphyria [11] and are more resistant to hepatic protoporphyria [50], which are caused by some iron-chelating drugs. In our congenic model, the differences in hepatic iron levels could not be explained by variations in known iron-related genes and thus, the novel modifier identified in this region may confer predisposition to these iron-related disorders in different mouse strains.

In summary, we validated and fine-mapped the QTL on Chr16 controlling hepatic iron accumulation. Using sequencing data and expression profile analyses, we identified three strong candidates, Sidt1, Boc, and Spice1, which had non-synonymous variations. This study could guide future work for identification of a novel genetic modifier involved in iron metabolism.

## Supporting Information

Figure S1 **Measurements of non-heme iron content of organs including heart, kidney, lung, and pancreas of original Chr16 congenic mice (n> = 5 for each group, 8 weeks).** Data represent mean ± SEM, N.S: no significance.
(TIF)

Figure S2 **Measurement of liver and spleen non-heme iron contents of congenic line 5, line 6, and line 7.** Data represent mean ± SEM, N.S: no significance, n = 5 for each group.
(TIF)

Figure S3 (A) Measurement of liver heme iron contents of Chr16 congenic line 3. (B) Relative mRNA expression of *Fth1*, *Sod1* and *Gpx4* in congenic mouse liver. (C) Relative mRNA expression of *IL-6*, *TNF-α* and *IL-1β* in Chr16 congenic mouse liver and spleen. (D) Relative mRNA levels of Fpn1 and TfR1 in congenic spleen. Expression of indicated genes is reported as relative expression levels using *β-actin* as an internal control in each group. The Chr16^s/s^: Chr16*^b/b^* ratios indicate relative expression levels of the Chr16*^s/s^* group normalized to the Chr16*^s/s^* group, which was defined as 1.0. n>5 for each group. Data represent mean ± SEM, N.S: no significance. * P<0.05, ** P<0.01.
(TIF)

Figure S4 **Conservation of the non-synonymous variations detected in genes** ***Boc*** **,** ***Sidt1*** **, and** ***Spice1*** **across species.** The position of each fragment is indicated by the numbers on the right and sources of sequences are shown on the left. The alignments were performed by the Clustal W Method. The black shading indicates residues that match the consensus.(A) *Boc* of C57BL alleles has unique Ser450 insertions, a polymorphism not previously reported. (B) B*oc* also has a Q1051R variation at a conserved residue. (C) *Sidt1* has a P172R variation and the residue is conserved between humans and rodents. (D) *Spice1* shows an R708S variation at a poorly conserved residue.
(TIF)

Figure S5 **Relative mRNA expression of candidate genes in Chr16 congenic mouse intestine (A) and spleen (B).** The Chr16^s/s^: Chr16*^b/b^* ratios indicate relative expression levels of the Chr16*^s/s^* group normalized to the Chr16*^s/s^* group, which was defined as 1.0. (C) Relative mRNA expression of candidate genes in mice liver treated with iron rich diet. The ratios represent the relative expression values normalized to control group, which was defined as 1.0. *β-actin* was as an internal control in each group. n> = 5 for each group. Data represent mean ± SEM, N.S: no significance. * P<0.05, ** P<0.01.
(TIF)

Figure S6 **Relative mRNA expression of iron related genes in mouse liver overexpressed the indicated candidate genes through hydrodynamic transfection.** n = 5 male mice for each group, *β-actin* was used as an internal control and data was represent as ratios of pLIVE control relative to candidate genes group. Data represent mean ± SEM, N.S: no significance.
(TIF)

Table S1 **Sequences of genotyping primers.**
(DOCX)

Table S2 **Sequences of oligonucleotide primers for Real-time PCR.**
(DOCX)

Table S3 **Interval candidate gene accession numbers.**
(DOCX)
