# Supplementary figures and images for: Fine-Mapping and Genetic Analysis of the Loci Affecting Hepatic Iron Overload in Mice
Source: PLoS One. 2013 May 10;8(5):e63280. doi: 10.1371/journal.pone.0063280 (PMC3651197; doi:10.1371/journal.pone.0063280)

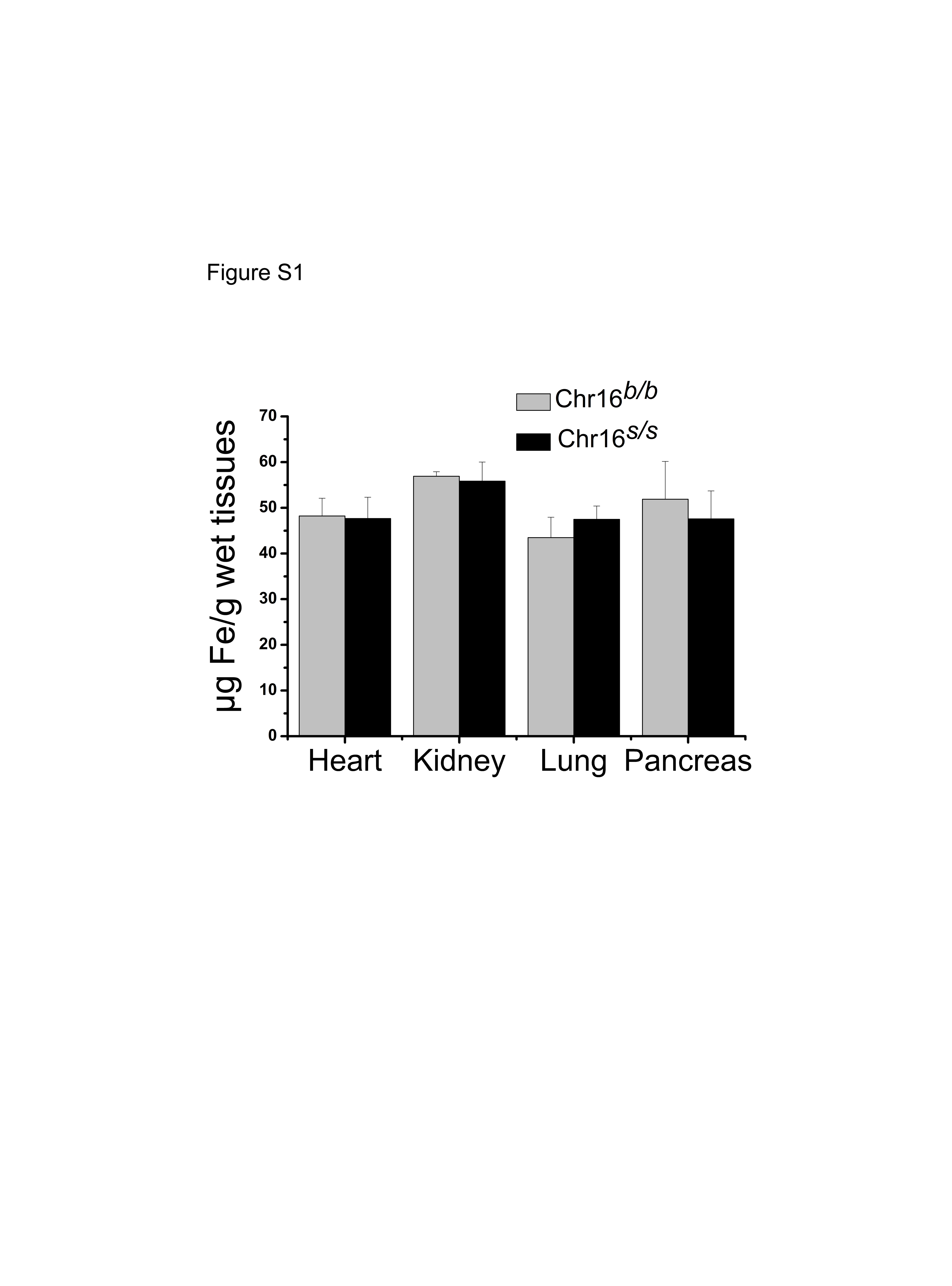

Supplement: Figure S1 — Measurements of non-heme iron content of organs including heart, kidney, lung, and pancreas of original Chr16 congenic mice (n> = 5 for each group, 8 weeks). Data represent mean ± SEM, N.S: no significance. (TIF) [file pone.0063280.s001.tif]

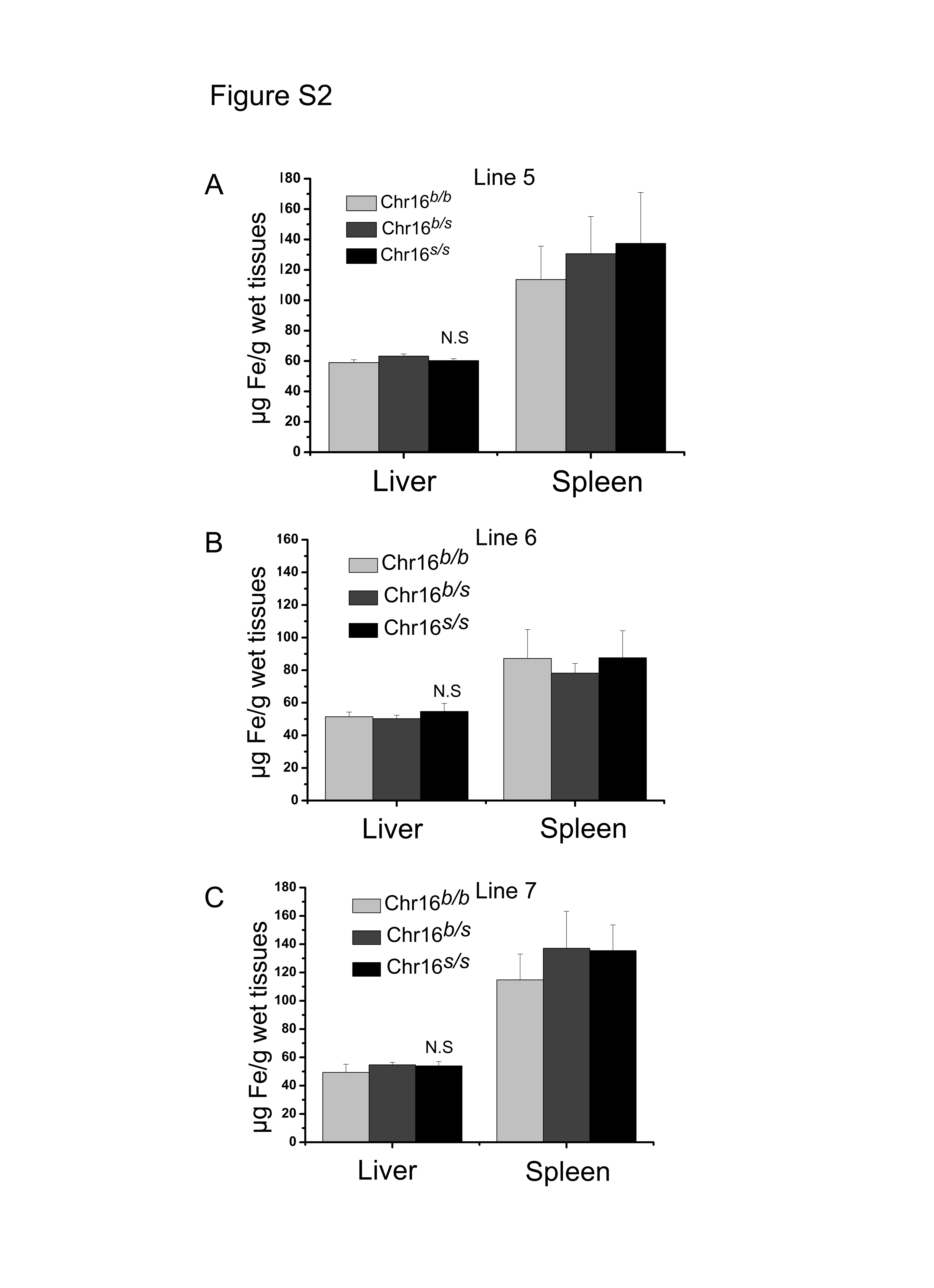

Supplement: Figure S2 — Measurement of liver and spleen non-heme iron contents of congenic line 5, line 6, and line 7. Data represent mean ± SEM, N.S: no significance, n = 5 for each group. (TIF) [file pone.0063280.s002.tif]

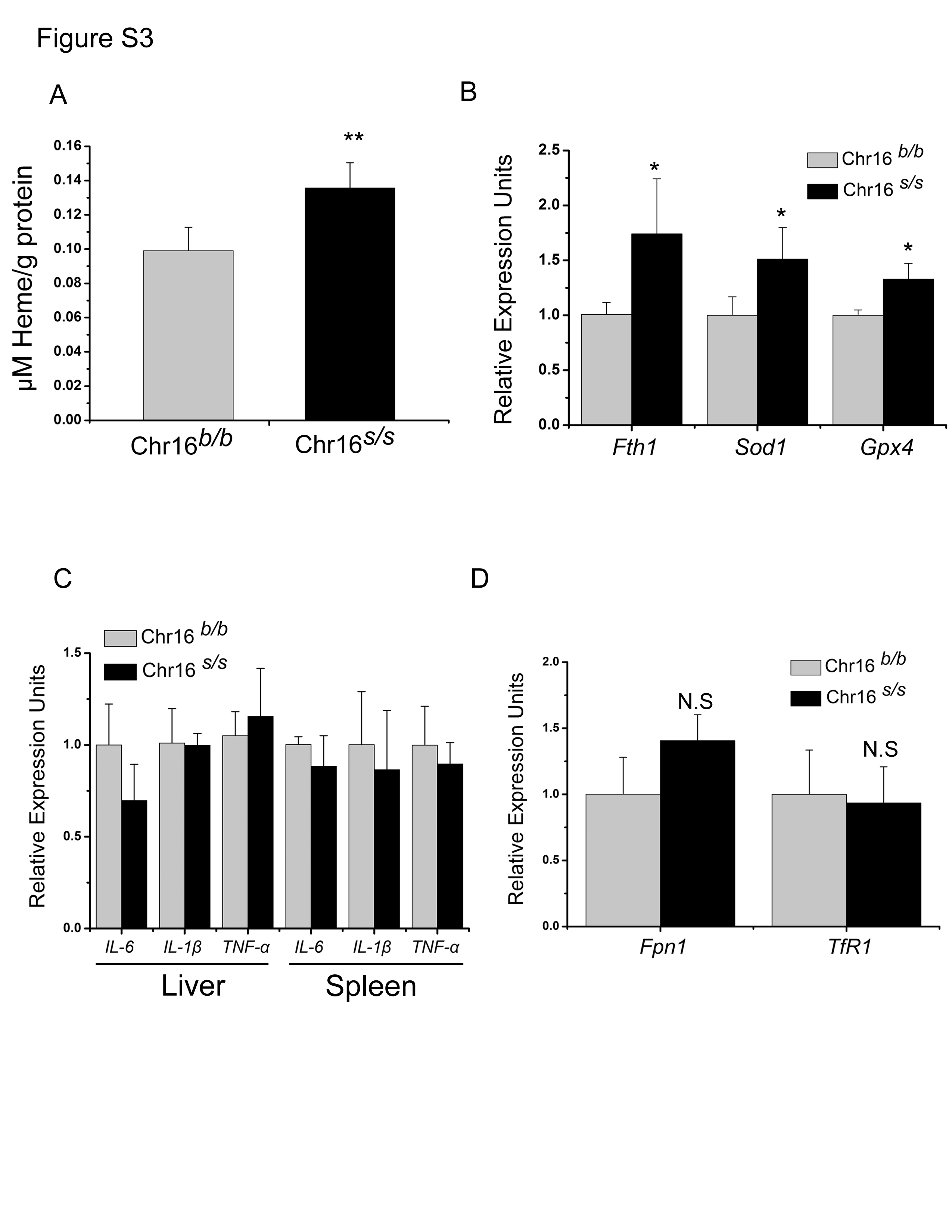

Supplement: Figure S3 — (A) Measurement of liver heme iron contents of Chr16 congenic line 3. (B) Relative mRNA expression of Fth1, Sod1 and Gpx4 in congenic mouse liver. (C) Relative mRNA expression of IL-6, TNF-α and IL-1β in Chr16 congenic mouse liver and spleen. (D) Relative mRNA levels of Fpn1 and TfR1 in congenic spleen. Expression of indicated genes is reported as relative expression levels using β-actin as an internal control in each group. The Chr16s/s: Chr16b/b ratios indicate relative expression levels of the Chr16s/s group normalized to the Chr16s/s group, which was defined as 1.0. n>5 for each group. Data represent mean ± SEM, N.S: no significance. * P<0.05, ** P<0.01. (TIF) [file pone.0063280.s003.tif]

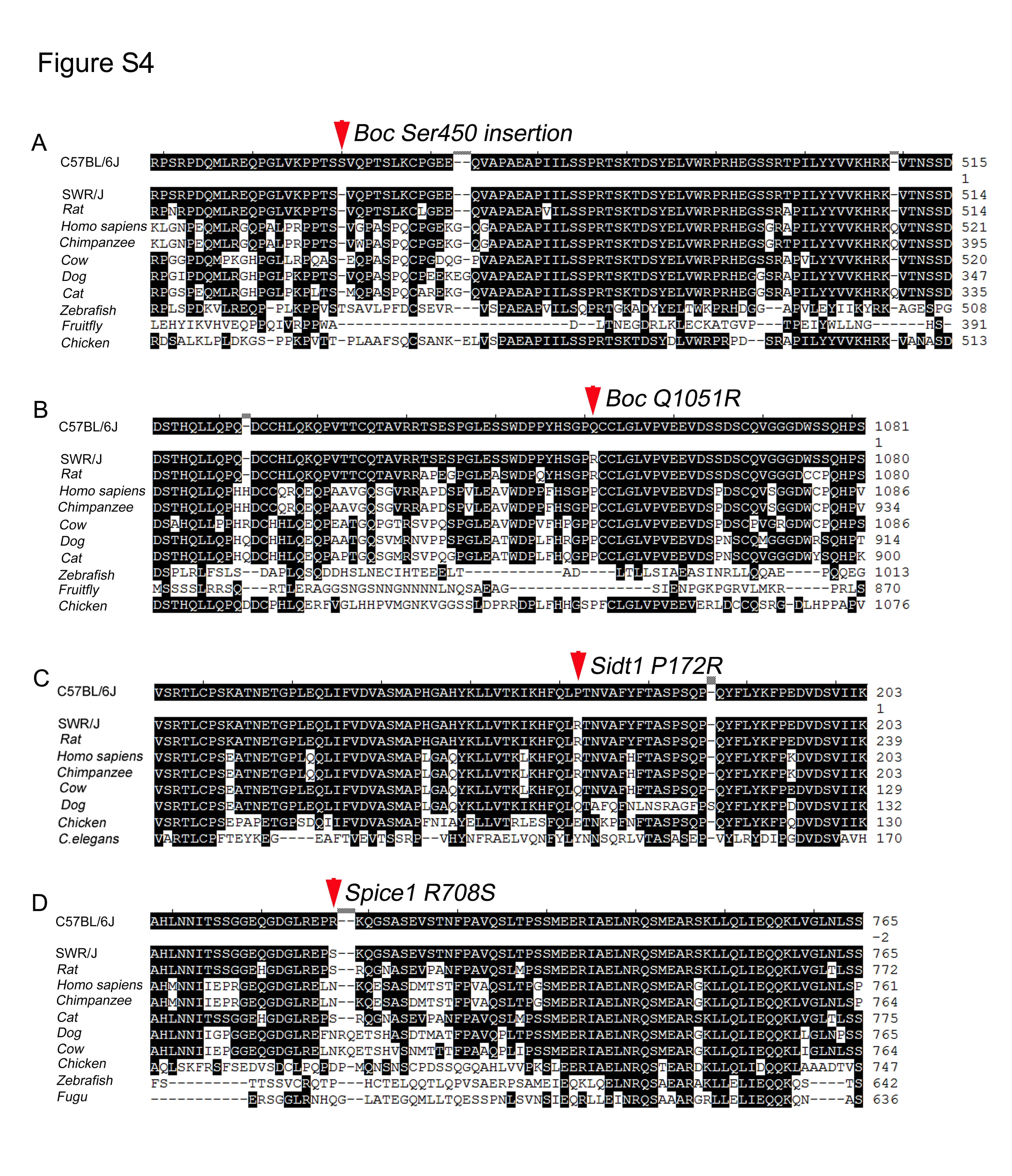

Supplement: Figure S4 — Conservation of the non-synonymous variations detected in genes Boc , Sidt1 , and Spice1 across species. The position of each fragment is indicated by the numbers on the right and sources of sequences are shown on the left. The alignments were performed by the Clustal W Method. The black shading indicates residues that match the consensus.(A) Boc of C57BL alleles has unique Ser450 insertions, a polymorphism not previously reported. (B) Boc also has a Q1051R variation at a conserved residue. (C) Sidt1 has a P172R variation and the residue is conserved between humans and rodents. (D) Spice1 shows an R708S variation at a poorly conserved residue. (TIF) [file pone.0063280.s004.tif]

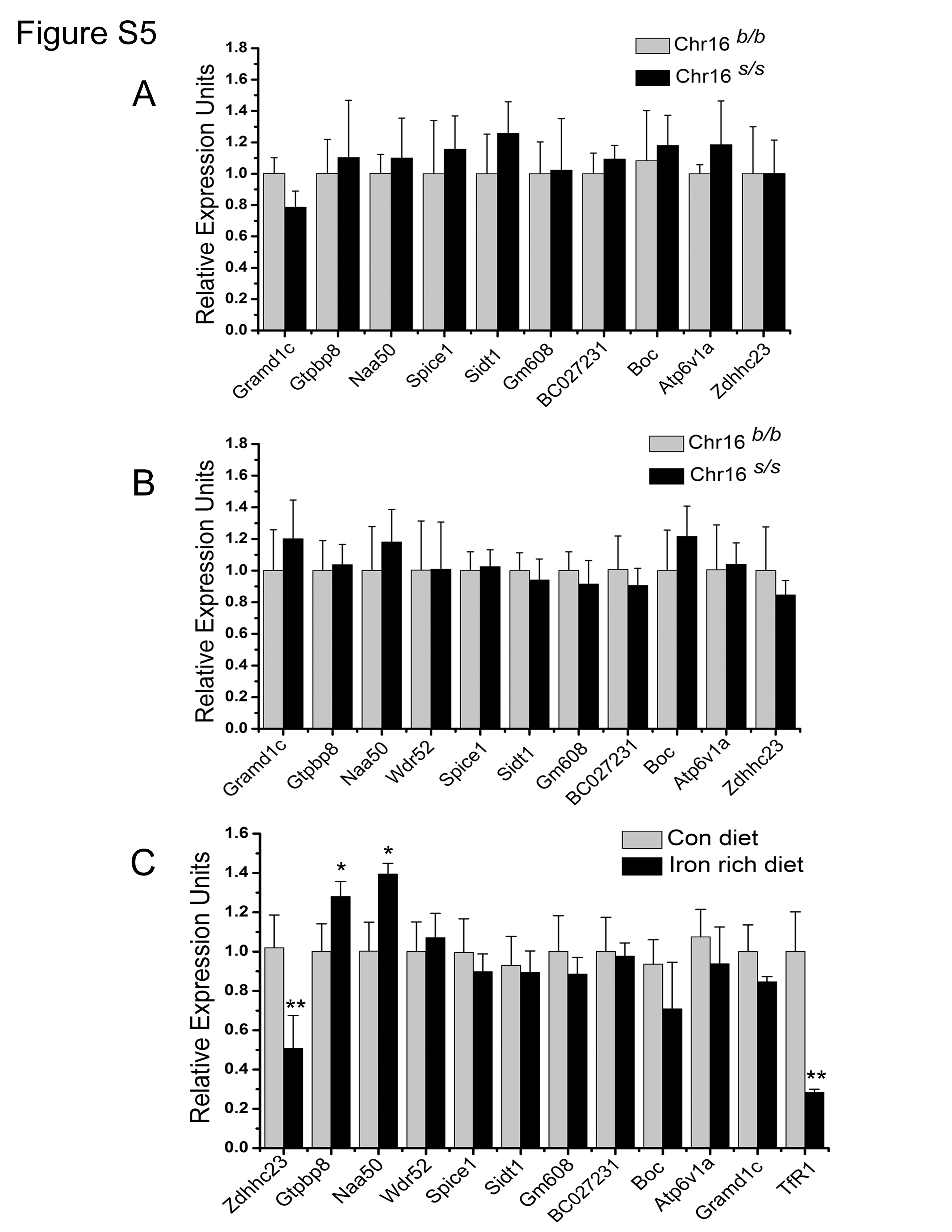

Supplement: Figure S5 — Relative mRNA expression of candidate genes in Chr16 congenic mouse intestine (A) and spleen (B). The Chr16s/s: Chr16b/b ratios indicate relative expression levels of the Chr16s/s group normalized to the Chr16s/s group, which was defined as 1.0. (C) Relative mRNA expression of candidate genes in mice liver treated with iron rich diet. The ratios represent the relative expression values normalized to control group, which was defined as 1.0. β-actin was as an internal control in each group. n> = 5 for each group. Data represent mean ± SEM, N.S: no significance. * P<0.05, ** P<0.01. (TIF) [file pone.0063280.s005.tif]

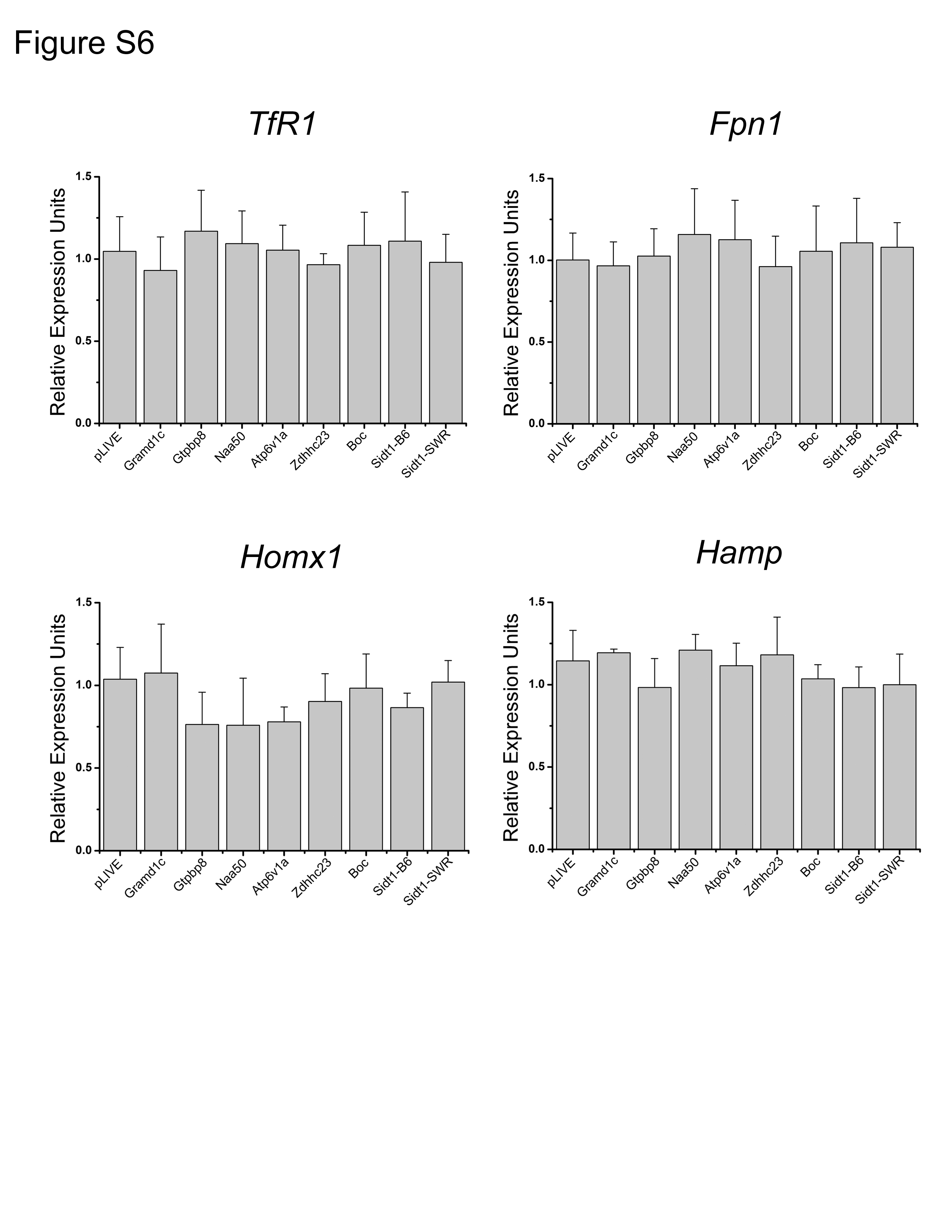

Supplement: Figure S6 — Relative mRNA expression of iron related genes in mouse liver overexpressed the indicated candidate genes through hydrodynamic transfection. n = 5 male mice for each group, β-actin was used as an internal control and data was represent as ratios of pLIVE control relative to candidate genes group. Data represent mean ± SEM, N.S: no significance. (TIF) [file pone.0063280.s006.tif]
