# Supplementary material for: Fine-Mapping and Genetic Analysis of the Loci Affecting Hepatic Iron Overload in Mice
Source: PLoS One. 2013 May 10;8(5):e63280. doi: 10.1371/journal.pone.0063280 (PMC3651197; doi:10.1371/journal.pone.0063280)
Supplement: Table S1 — Sequences of genotyping primers. (DOCX) [file pone.0063280.s007.docx]

**Table S1** Sequences of genotyping primers

| Chromosome 16 | **Position(Mb)** | **Left** | **Right** |
| --- | --- | --- | --- |
| D16Mit13 | 39.1 | TTAGAACTCAGTAAGCTCTC | CTTAAACAGGCACNAATCCCATT |
| D16Mit38 | 41.4 | GGGAGGTAGACTAGCAACATGG | AATGAAATACACATACCCACATGC |
| D16Mit125 | 42.3 | CAGAGATTACAAGCATACATCTTAGC | CAAACAACAAAACACATTCAACTC |
| D16Mit41 | 42.87 | TGGAGAAGAGGAATAAGGGATG | CCCAGCCCCAGTAAATGTAA |
| D16Mit39 | 43.45 | CAGCGTCTAAATGGATTGAGTG | AGTGTCAGACCTTTTAAGGACACA |
| D16Mit198 | 44.18 | CTGTAAATGATCCTTAACATATGAATG | AGTTCAATGCCTGGGTATGG |
| D16Mit379 | 44.3 | ATTTAAATATCTCAAACTCCTCCCG | GACCAGCATCAAATGGACG |
| Boc-In4-P6 | 44.5 | CCATCTGAAGCTCTGTGCAT | TCTCCCACATGGACTTCTGA |
| Cd200R1-In1-P2 | 44.7 | TCTCCATTGACTGGCTTTGA | TCAGTTACTTGTGCCAACTGC |
| D16Mf1 | 44.9 | ATCAACCAACCACAGGC | AACCCACACAGAACTTG |
| D16Mit84 | 45.3 | GAGCATCATCCTGCTGATCA | AGTTTAAGCAAAGATGAGGGAGG |
| D16Mit126 | 45.9 | TTGTTCACAAAAACTGGGAGG | AGACTCCAACCTTCTTACGTTCC |
| D16Mit61 | 48.3 | CCCATTCCCCAAACTTGAAT | TCCTGTCTCAAAACAATAAGAGAGA |
| D16Mit30 | 53.8 | GTGCACATACATACCACAGCG | TCACTGCAGGGAGGTTCAG |
| D16Mit64 | 57.3 | TACCATGATCAGTCCAAAGGC | ACTTAAGGTTGTCCTGTGGGG |
| D16Mit185 | 60.4 | AAAAAAAAGGAAAATATCAAGTCACA | AAGTGATTTTCTATTGTTCTGGCA |
| D16Mit216 | 61.7 | CAGGGATGATTCAGAATATTTATGG | AAATCCAGCAGTTCCTTTATGG |
| Chromosome 7 | **Position(Mb)** | **Left** | **Right** |
| D7Mit68 | 125.1 | CTCCCACACAGGGTCTTTGT | GATACCCAAAGTACACCTCTGTCA |
| D7Mit105 | 128.3 | AGCAAAGTAAGGCAGACTTTGG | AGGAGAGGCAGAACATGGAA |
| D7Mit71 | 130.82 | CCACCTGGAATACATGTAACCC | TTTGCTTATTCTAAGCCCAAGC |
