# Supplementary material for: Fine-Mapping and Genetic Analysis of the Loci Affecting Hepatic Iron Overload in Mice
Source: PLoS One. 2013 May 10;8(5):e63280. doi: 10.1371/journal.pone.0063280 (PMC3651197; doi:10.1371/journal.pone.0063280)
Supplement: Table S2 — Sequences of oligonucleotide primers for Real-time PCR. (DOCX) [file pone.0063280.s008.docx]

# Table S2 Sequences of oligonucleotide primers for Real-time PCR

| **Gene** | **Forward primer** | **Reverse primer** |
| --- | --- | --- |
| *Actin* | AAATCGTGCGTGACATCAAAGA | GCCATCTCCTGCTCGAAGTC |
| *Hamp* | GCACCACCTATCTCCATCAACA | TTCTTCCCCGTGCAAAGG |
| *Atp6v1a* | AGCTGCCCGCCAATCAC | CGGGAAAAGGGCATCGA |
| *BC027231* | TGGTGGAGGTGGTGCTGAT | CCAGCAAGCGCAGTAACAAC |
| *Boc* | CACCATGGATGAACGTGACTTG | CAGAGCATCATCAGAGCCATTT |
| *Gtpbp8* | TTGAAGGAGATTCGGCATTAAGA | ACCCCCAGCGCATTTGT |
| *Gm608* | TGGCGAGTCAGATCACACCTAA | GTGCAAGGCCATCTCCATGT |
| *Spice1* | CACCGGGCAACTCCTGAA | GCACCAATGCTCTATTCTTTGACTT |
| *Wdr52* | GGGAAGGCAGCCTCATCAA | TGGAACCGCTGTGACAAGAC |
| *Naa50* | CCGGAAGTTTGGCTTTGAGA | CGTCTGCAGGCTCTATCCTCTT |
| *Sidt1* | CATGTCTGCCCTAATTACTCCAACT | GAGGCCAGCGATCATGTACA |
| *Zdhhc23* | ACAGCCCTGCCAAAGTGAAG | CCGGGCGGGTCGTACTA |
| *Gramd1c* | ACCCGCTAACTGGGAAGTATACTG | TGTGCTTCCCGGCTCTCTT |
| *Fpn1* | GTCGGCCAGATTATGACATTTG | ATTCCAACCGGAAATAAAACCA |
| *Hmox1* | CACTTCGTCAGAGGCCTGCTA | GTCTGGGATGAGCTAGTGCTGAT |
| *TfR1* | CTCAGTTTCCGCCATCTCAGT | GCAGCTCTTGAGATTGTTTGCA |
| *TfR2* | TCCAAGAAACCCAGAGACCTGTT | CCGAGTCCTGAGTGGGAAGA |
| *Hfe* | TGTGAGGTGCATGAAGACAACAG | TCTTGCCCGTCATAACCATATCT |
| *Hjv* | CCAGGCTGAGGTGGACAATC | GTCGGTCGCCCCCATT |
| *Bmp6* | AACCTTTCTTATCAGCATTTACCA | GTGTCCAACAAAAATAGGTCAGAG |
| *Fth1* | AAGATGGGTGCCCCTGAAG | CCAGGGTGTGCTTGTCAAAGA |
| *Ftl1* | CGGGCCTCCTACACCTACCT | CCCTCCAGAGCCACGTCAT |
| *Heph* | TGTGAGGTGCATGAAGACAACAG | TCTTGCCCGTCATAACCATATCT |
| *Sod1*  *Gpx4*  *DMT-1* | ACCAGTGCAGGACCTCATTTTAAT  TCCGAGTTCCTGGGCTTGT  GCGGCCAGTGATGAGTGAGT | TCTCCAACATGCCTCTCTTCATC  GCTGAGAATTCGTGCATGGA  ATGCCACCGGCAATCCT |
