# Supplementary material for: Fine-Mapping and Genetic Analysis of the Loci Affecting Hepatic Iron Overload in Mice
Source: PLoS One. 2013 May 10;8(5):e63280. doi: 10.1371/journal.pone.0063280 (PMC3651197; doi:10.1371/journal.pone.0063280)
Supplement: Table S3 — Interval candidate gene accession numbers. (DOCX) [file pone.0063280.s009.docx]

**Table S3** Interval candidate gene accession numbers

| **Official Symbol** | **Accession number** | **Official Full Name** |
| --- | --- | --- |
| *Zdhhc23* | NM_001007460 | zinc finger, DHHC domain containing 23 |
| *Gramd1c* | NM_001172107 | GRAM domain containing 1C |
| *Atp6v1a* | NM_007508 | ATPase, H+ transporting, lysosomal V1 subunit A |
| *Naa50* | NM_028108 | N(alpha)-acetyltransferase 50, NatE catalytic subunit |
| *Gm608* | NM_001029889 | predicted gene 608 |
| *Sidt1* | NM_001159419 | SID1 transmembrane family, member 1 |
| *Spice1* | NM_144550 | spindle and centriole associated protein 1 |
| *Wdr52* | NM_001033247 | WD repeat domain 52 |
| *Boc* | NM_172506 | biregional cell adhesion molecule-related/down-regulated by oncogenes (Cdon) binding protein |
| *BC027231* | NM_145972 | cDNA sequence BC027231 |
| *Gtpbp8* | NM_001159329 | GTP-binding protein 8 (putative) |
